# Radiation dose evaluation of pediatric patients in CT brain examination: multi-center study

**DOI:** 10.1038/s41598-021-84078-z

**Published:** 2021-02-25

**Authors:** Fotso Kamdem Eddy, Samba Odette Ngano, Fotue Alain Jervé, Abogo Serge

**Affiliations:** 1 Unité de Recherche de la Matière Condensée, d’Electronique et de Traitement du Signal, Department of Physics, Faculty of Science, University of Dschang, Dschang, Cameroon; 2 Department of Radiography, Yaoundé General Hospital, Yaoundé, Cameroon; 3 Department of Radiology, National Social Insurance Fund Hospital, Yaoundé, Cameroon

**Keywords:** Biophysics, Cancer, Physics

## Abstract

There is currently no Pediatric Regulatory Diagnostic Reference Level (DRL) in Cameroon to standardize protocols in hospitals. France, a European country, has DRL allowing them to optimize their examination protocol. For the sake of radiation protection, we have proposed to evaluate the dose and acquisition parameters delivered to our pediatric patients to optimize the protocols used. We also compared the 75th percentile values of dose parameters by acquisition between the three hospitals to Diagnostic Reference Level (DRL) of France. In this retrospective and evaluative multicenter study, a total of 320 patients who had at least one cranial CT scan were enrolled from three medical centers. The CT acquisition parameters including tube potential (kV), tube current (mA), slice Thickness (T), spiral or sequential scanning techniques, volume CT dose index (CTDIvol), and dose length product (DLP) were analyzed. CTDIvol values in our centers were found up to 17.42%, 46.01%, 21.56% respectively for children aged 1–4 higher than values of France's DRL. For those aged 5–9, we obtained 44.58%, 43.15%, 42.21% respectively. In addition, for children aged 10–14 there are also up to 47.73%, 44.11%, 46.39% respectively higher than values of France's DRL. It is similary for DLP values. The study showed a significant dosimetric overshoot compared to the France’s DRL and prompted us to make corrections to the protocols used and to a more rigorous monitoring of the principles of radiation protection and optimization rules in pediatric computed tomography in our hospitals. Our results have led us to make changes to our protocols which are the subject of a new dosimetric evaluation. The development of DRL for improving the pediatric CT scan in our country is necessary to optimize our protocols. Our results have led us to make changes to our protocols which are the subject of a new dosimetric evaluation. It would be necessary to set up a quality control structure in Cameroon and their applications in current practice.

## Introduction

The International Commission on Radiological Protection (ICRP) introduced the Diagnostic Reference Level (DRL) as the tool for optimizing dose management in medical imaging procedures^1–4^. In Cameroon, there is not yet a regulatory pediatric DRL. In France, the good practice of radiation protection is regulated by texts and laws which were issued in 2004 and updated in 2011^5^. They specify that on the CT scanner, the dosimetric data (volume CT dose index (CTDIvol), and dose length product (DLP)), relating to each examination and each acquisition, must be included in the report issued to patients. They also specify that the responsible radiologist, whether in a private or hospital structure, is required to carry out a dosimetric, annual and comparative assessment of the Diagnostic Reference Levels (DRL) in force, of the doses delivered to patients (DLP and CTDIvol) during standard practice scanners.

France has pediatric DRL^6,7^ which are updated regularly (DRL France 2019) by the Institute for Radiation Protection and Nuclear Safety (IRSN) and the France Society For Pediatric and prenatal Imaging (SFIPP). International norms^8–10^, European^11^, France^6,7,12^ and regulatory frameworks^13,14^ require that the equipment, accessories and procedures be adapted to the practice of pediatric imaging. Any examination must be justified by its diagnostic contribution in relation to irradiation. Its performance must be optimal, that is to say in accordance with the ALARA principle, As Low As Reasonably Achievable, and the doses delivered must be regularly evaluated to compare with the diagnostic reference levels, these must not be exceeded without justification.

Pediatric radiology requires specialized equipment, specific precautions and specialized knowledge of ionizing radiation. Studies published in this country propose to protect the eyes, thyroid and gonads in pediatric tomodensitometry with the leaded apron^15^ during a CT scan. They also propose the dose optimization in computer tomography pediatric cranial scans^16^ and how to reduce absorbed doses of radiosensitive organs of children exposed to ionizing radiation on adult scanners^17^. Another research proposes strategies to optimize the exposure of pediatric patients on adult scanners^18^.

Medical imaging plays a crucial role in the treatment of these children. This is not an easy task for developing countries. Some developing countries still use older generation CT scanners due to multiple handicaps like poverty, underdevelopment, the reduced number of radiologists and hospitals. The purpose of this study is to assess the doses received by pediatric patients in large hospital units in Cameroon and to compare them with the France’s DRLs.

The need to minimize the doses received during the examinations is essential in diagnostic radiology and even more so in pediatric CT. Indeed, patient-specific dosimetry is of significant interest in pediatric cranial applications, as radiation sensitivity is outstandingly higher compared to adults. This is because children have a higher risk to develop cancer compared to adults receiving the equivalent dose^19^. Giving the possible risk of X-ray radiation to pediatric patients, low-dose CT has aroused considerable interest in the biomedical imaging field^20^. The results and recommendations of this work will be used to help professionals involved in radiology in the estimation of doses delivered to patients during CT examinations, to educate medical staff in hospitals and the population on the quantity of doses delivered and to meet requirements set out in European Directive 97–43^21^ on the radiological protection of patients resulting from publication 73 of the ICRP on "Radiological Protection and Safety in Medicine"^22^ which requires member states to estimate the doses received by the population.

## Methods

### Study population

The local Ethical Committee of Regional Center for Medical Imaging of the West (CRIMO) institutional review board approved this retrospective multi-center (Hospital 1, Hospital 2 and Hospital 3) study with the protocol number of Ydé 19/02. Method was carried out in accordance with relevant guidelines and regulations. A total of 320 patients of both sexes who had at least one cranial CT scan between August 2019 to March 2020 were enrolled from three medical centers. The patients formed four age groups: < 1 year, 1 to 4 years, 5 to 9 years and 10 to 14 years. The clinical reasons exploited were mainly cases of convulsion, headache, trauma and tumor. Patients without detailed radiation dose reports were excluded from the study. Verbal informed consent from the patients’ legal guardian was obtained for all patients.

### CT Technique and dose analysis

The study data were obtained from three different medical centers and radiology information systems using a preformatted data form (age, sex, type of CT scanner, diagnosis on CT, number of cranial CT scans and the CT acquisition parameters including tube potential (kV), tube current (mA), slice thickness (cSL (mm)), spiral or sequential CT scanning techniques and CT dose products, volume CT dose index (CTDIvol (mGy)), dose length product (DLP (mGy.Cm)). The participating blinded radiologists obtained the parameters relevant to radiation dose from the scan protocol generated by the three different CT systems (1 Neusoft 64, 1 Hitachi 16 and 1 Toshiba 128), from the three centers after each cranial CT. Mean and cumulative radiation dose values of patients from cranial CT scans performed during the study inclusion period were calculated using CTDIvol and DLP. Dose values of patients were compared; the variability of CT acquisition parameters and effect of CT acquisition parameters on emitted radiation doses were interrogated.

### Statistical analysis

Fisher test was used in the analysis of contingency tables and Kolmogorov–Smirnov test was used to determine whether continuous variables were normally distributed. The significance of the difference in terms of continuous variables in which parametric test assumptions were provided between the groups was evaluated by Student's t-test. All the data were analyzed on the Microsoft Excel 2016 calculation software. For all the exams and for a single acquisition, we collected the CTDIvol and DLP. We calculated the values ​​of the 75th percentile in order to compare them with the France ‘s DRL.

## Results

The age of patients, type of CT scanners and acquisition parameters of CT examinations are presented in Table 1. 320 patients participated in this study (80 for age < 1, 80 for age 1–4, 80 for age 5–9 and 80 for age 10–14).

**Table 1:** Protocol acquisition parameters for the three scanners used.

Age (Year)	Hospitals	kV mean/median (min–max)	mA mean / median (min–max)	Slice thickness (mm)mean / median (min–max)
< 1	Hospital 1	108.94/100 (100–120)	193.45/150 (149–360)	3.67/1.25 (1.25–5)
	Hospital 2	118/120 (100–120)	131.81/122.3 (76–222.8)	1.25/1.25 (1.25–2.5)
	Hospital 3	120/120 (120–120)	150 (150–150)	1.25 (1.25–1.25)
1–4	Hospital 1	108.94/100 (100–120)	217.8/187.85 (150–400)	4.57/5 (1–5)
	Hospital 2	118/120 (100–120)	126.6/129 (75.8–188.3)	1.25/1.25 (1.25–2.5)
	Hospital 3	120/120 (120–120)	–	–
5–9	Hospital 1	108.94/100 (100–120)	270.64/300 (116.1–360)	3.56/5 (1–5)
	Hospital 2	118/120 (100–120)	148.83/138 (84–248)	1.25/1.25 (1.25–2.5)
	Hospital 3	120/120 (120–120)	280 (280–280)	1.25 (1.25–1.25)
10–14	Hospital 1	108.94/100 (100–120)	303.33/300.6 (229.6–360)	2.1/1.25 (1.25–5)
	Hospital 2	118/120 (100–120)	176.81/167.3 (84–398)	1.25/1.25 (1.25–2.5)
	Hospital 3	120/120 (120–120)	–	–

The high voltages (kV) used ranged from 100 to 120. In comparison, the mean of tube potential of CT examinations per patient in Hospital 3 (120) was highest than Hospital 1 (108.94) and Hospital 2 (118) (Table 1). Mean mA and slice thickness values in Hospital 2 and Hospital 3 were significantly lower than Hospital 1. The CT scanner presented in post acquisition a dosimetric report on which appeared the delivered doses expressed in CTDIvol (Volume CT Dose Index) and DLP (Dose Length Product) for each acquisition and for the whole of the examination.

The details of the dose reports are provided in Table 2. Table 2 shows the comparison of CTDIvol and DLP of each of the hospitals with the DRL of France^21^ 2019. Of the three CT scanners used, only the Hitachi CT scanner had its shielded covers to protect the radiosensitive organs of children contained in long scan lengths. The dosimetric indicators noted (CTDIvol and DLP) in the three hospitals studied exceeded the France ‘s DRL in all cases (Tables 2). With regard to CTDIvol, for children aged 1–4, the values ​​of the three hospitals exceed the DRL by 17.42%, 46.01%, 21.56% respectively. For those aged 5–9, we have 44.58%, 43.15%, 42.21% respectively. In addition, for children aged 10–14 there is also an excess of 47.73%, 44.11%, 46.39%.

**Table2:** Dosimetric parameters recorded on the three CT scanners used compared to the Diagnostic Reference Levels (DRL) of France 2019.

Ages (Year) Hospitals	CTDIvol (mGy) 75th percentile (extreme values)				DLP (mGy.Cm) 75th percentile (extreme values)			
	< 1	1–4	5–9	10–14	< 1	1–4	5–9	10–14
Hospital 1	15.32 (12.1–54.8)	24.22 (8.6–49.4)	39.7 (14.2–54.8)	49.75 (27.8–54.8)	311.52 (194.4–1172.8)	456.9 (183.3–1075.9)	864.72 (136.7–1183.3)	1021.92 (376–1263.2)
Hospital 2	34.3 (14.6–62.5)	37.05 (14.9–43.1)	38.7 (17.7–51.8)	46.52 (19.6–61.9)	829.9 (262.9–1266.2)	906.65 (349.1–1129.5)	984.8 (417.9–1340.1)	990.82 (280.4–1949.8)
Hospital 3	17.1 (17.1–47)	25.5 (17.1–51.5)	38.07 (17.1–53.9)	48.5 (17–67.4)	379.35 (161.5–922.6)	504.75 (181.7–1371)	945.77 (249.6–1319.4)	1155.2 (461.2–1761.6)
DRL	–	20	22	26	–	320	360	470

Similarly DLP values ​​of the three hospitals for children aged 1–4 years were significantly higher than the France’s DRL by 29.29%, 64.70%, 36.60% respectively. 58.36%, 62.05% and 61.93% for those 5–9 years and 54%, 52.56%, 59.31% for children 10–14 years.

For the first two age groups, the CTDIvol of the Hospital 2 were significantly higher than Hospital 1 and Hospital 3. The DLP were significantly higher for the first three age groups. The CTDIvol values of Hospital 1 were significantly higher than Hospital 2 and Hospital 3 ​​for children 5–9 and 10–14 years. The DLP values of Hospital 3 were significantly higher than others Hospitals for children 10–14 years (Table 2).

## Discussion

The major findings of our study were as follows; CTDIvol and DLP values in our centers were found significantly higher than values of France's DRL. Furthermore, dose parameters were significantly variable between centers due to the incompatible CT protocols which would be standardized.

In this retrospective multi-center based study, we also compared the CT acquisition parameters and dose products between different centers and CT vendors in our country. Variable dose values and applied CT acquisition parameters of the three centers were more variable than expected, which denotes the importance of standardization of absorbed dose.

However, pediatric examination can be performed with low kV and mAs values^17^. Radiation exposure should always be managed in the ALARA (As Low As Reasonably Achievable) principle. Increasing the gantry rotation speed and the slice collimation (cSL) by changing detector configuration can decrease the radiation dose without significantly lowering the contrast resolution and yield acceptable CT image quality^23^. In clinical CT, Zacharias et al. told that it is challenging, if not impossible, to determine a fixed set of acquisition parameters that will provide “necessary” image quality^24^. So, CT protocols should be reviewed regularly to ensure that image quality and dose are being optimized^24^.

In addition, our results showed a positive correlation between kV and CTDIvol. In our opinion, this unexpected correlation would be due to the high kV use in our hospitals. Our study also revealed a significant difference between acquisition parameters of imaging centers.

The present study emphasizes the need for optimization of cranial CT protocols because ionizing radiation dose of cranial CTs from three CT scanners were significantly different. Comparison of CT radiation doses in terms of CTDIvol and DLP yielded markedly higher dose values of Hospital 2 (DLP: 1949.8) than the other two centers (DLP: 1761.6 and 1263.2 in Hospital 3 and Hospital 1, respectively). Radiation dose reduction principles consist of appropriateness criteria for optimization of imaging techniques. Although the extreme values of doses of Hospital 1 was low, an increased radiation dose exposure in this center was incompatible with the ALARA principle due to unoptimized CT acquisition parameters.

Optimization of CT acquisition parameters is important, especially in clinical circumstances that need repetitive CT scans. This study revealed that ionizing radiation dose exposure value of CT scans was correlated with kV, mAs, tube rotation time, and slice thickness values in CT protocols. Decreasing tube potential (kV) and tube current (mA) values and increasing tube rotation time results in reduced radiation dose^25,26^. Most medical imaging technicians do not understand the impact of acquisition parameters on dose reduction. They do not know that a brain CT scan can be substituted for an MRI to limit the exposure of patients. They should know that reducing the voltage below 120 kV for the skull and the tube current between 125 and 150 mAs has been shown to be effective in reducing the dose delivered by around 40% without altering the diagnostic quality of the images^26–28^. This is explained by the fact that most of them are not researchers and are not informed of new scientific discoveries in their field. We have modified the protocol by proposing a millimeter acquisition thickness (5 mm) in order to use the iterative reconstruction technique at less than 5 mm and a mA of less than 200. The dosimetric evaluation of new pediatric protocols is the subject of an ongoing study. Dose reduction techniques in these CT scanners are not generally used because the operators ignore their presence or because they do not know how to use it. Some manufacturers offer automatic dose modulation systems, AEC, Automatic Exposure Control, which further reduce the doses delivered^28^. Training in the subject must be set up.

In Cameroon, the national DRL are not established, either in pediatrics or for adults. Practices are not harmonized in all hospitals. But Moifo et al. have proposed A Pilot Study of Four Commonest CT-Protocols in Five Radiology Departments for the Diagnostic Reference Levels of Adults CT-Scan Imaging in Cameroon^29^. Cameroon does not have a quality control structure to verify the compliance of the radiation doses delivered to patients with the DRL. Dosimetric overshoots for the CT scans protocols in our daily practice were noted and we attributed these overshoots to unsuitable technical parameters on our protocols (kV, mA, cutting thickness, CTDI_Vol_ limitation of scan length, knowledge of the subject of radiation protection, dose reduction software, size of pediatric patients compared to adult patients, exposure of children on adult machines, mastery of the tool, choice of radiological parameters suitable for a type of examination).

The use of radiological protocols and procedures not suitable for pediatric examination leads to the use of large doses of radiation. In fact, our technicians do not rigorously and conscientiously respect the principles of radiation protection and the radiological and non-radiological protocols recommended by the international radiation protection commissions^30^. This exposes children to already very high doses in the case of a single acquisition can later lead to cancer if these children undergo another CT scan during their lifetime because the doses are cumulative^31^. A CT scan, given its particularly irradiating nature, should only be performed in a child if absolutely necessary^32^. MRI should be preferred whenever they are likely to provide the information sought and when they are available within a period compatible with the urgency of the situation. The need for a successful acquisition from the first pass is better on the CT scan than in conventional radiology.

Our study has several limitations. First, we were not able to find some of the CT acquisition parameters in the dose report of CT examinations, which limited to evaluate the effects of each parameter on CT doses. The second limitation was the absence of information about the CT scan history of these patients in other centers. The third limitation was the unavailability of different parameters such as tube filtration, iterative reconstruction technique, and detector configuration, all of which vary across vendors. We did not evaluate the effect of modification of these parameters on image quality.

## Conclusion

This work allowed to know the importance of dose reduction software, dose reconstruction techniques, the impact of acquisition parameters on the reduction of the dose absorbed to patients, in particular to pediatric patients. Our study revealed dosimetric exceedances compared to the 2019 DRL adopted for France. We had not noted these exceedances earlier because, in the absence of regulatory obligations, we do not carry out annual quality control evaluations periodically of the radiation doses delivered to our patients. Our results have led us to make changes to our protocols which are the subject of a new dosimetric evaluation. It would be necessary to set up a quality control structure in Cameroon and to create continuous training for medical and paramedical personnel in radiology in terms of knowledge of dose reduction software, dose reduction techniques and their applications in current practice.

## References

[1] International Commission on Radiological Protection Radiological protection in medicine. ICRP Publication 105. *Ann ICRP* **37**, 1–64 (2007). 10.1016/j.icrp.2007.10.003 18082557

[2] International Commission on Radiological Protection 1990. Recommendations of the International Commission on Radiological Protection. ICRP Publication 60. *Ann ICRP* **21**, 1–201 (1991). 2053748

[3] International Commission on Radiological Protection. Radiological protection and safety in medicine. ICRP Publication 73. *Ann ICRP* **26**, 1–47 (1996). 8911634

[4] International Commission on Radiological Protection. Diagnostic reference levels in medical imaging: review and additional advice. ICRP Supporting Guidance 2. *Ann ICRP* **31**, 33–52 (2001). 12685758

[5] JORF Arrêté du 24 octobre 2011 relatif aux niveaux de référence diagnostiques en radiologie et en médecine nucléaire. Paris, France (2012).

[6] SFIPP. Critères de qualité et optimisation des doses en scanographie chez l’enfant. Accès le 19 Mars 2020 sur http://www.sfipradiopediatrie.org/images/storie/docs_telechargement/guideprocedurestdmpedi06.pdf (2006).

[7] Brisse H, Aubert B (2009). Niveaux d’exposition en tomodensitométrie multicoupes pédiatrique : résultats de l’enquête dosimétrique SFIPP/IRSN 2007–2008. J. Radiol..

[8] IAEA. Radiation protection of patients, CT optimization. Accès le 19 Mars 2020 sur : https://rpop.iaea.org/RPOP/RPoP/Content/InformationFor/HealthProfessionals/1_Radiology/ComputedTomography/CTOptimization.htm (2011).

[9] Valentin J (2007). Managing patient dose in multi-detector computed tomography. Ann. ICRP.

[10] Geleijns, J. Optimisation of radiation protection for pediatric and adult patients in radiography and computed tomography, Proceedings Third European IRPA Congress 2010, Helsinki, Finland. pp. 2972–80 (2010).

[11] Conseil de l’union européenne (1997). Directive 97/43 EURATOM du Conseil du 30 juin 1997 relative à la protection sanitaire des personnes contre les dangers des rayonnements ionisants lors d’exposition à des fins médicales.

[12] Mabille M, Beauvais-March H, Rehel J-L, Kalifa G (2005). Évaluation des doses d’irradiation aux organes en scanographie pédiatrique. J. Radiol..

[13] SFR. Guide pratique à l'usage des médecins radiologues. Accès le 19 Mars 2020 sur http://www.sfrnet.org/sfr/societe/2-publications/publications-sfr/01Guides2009/index.pht ml (2009).

[14] IRSN. Rapport DRPH n°2010–15. Analyse des données relatives à la mise à jour des niveaux de référence diagnostiques en radiologie et en médecine nucléaire. Bilan 2007–2008. Paris, France (2010).

[15] Fotso KE, Samba ON, Fotue AJ, Fai LC (2020). Protection of the eyes, thyroid and gonads in pediatric tomodensitometry: use of the leaded Apron. AASCIT J. Health..

[16] Takam CA (2019). Dose optimization in computer tomography pediatric cranial scans. Open J. Radiol..

[17] Fotso KE (2019). Reduce absorbed doses and protection of radiosensitive organs of children exposed to ionizing radiation on adult scanners. Insights Med. Phys..

[18] Fotso KE, Samba ON, Abogo S, Fotue AJ (2020). Techniques to optimize the exposure of pediatric patients on adult scanners. AASCIT J. Health..

[19] Papadimitroulas P, Kostou T, Chatzipapas K (2018). A review on personalized pediatric dosimetry applications using advanced computational tools. IEEE Trans. Radiat. Plasma Med. Sci..

[20] Chen H, Zhang Y, Kalra MK (2017). Low-dose CT with a residual encoder-decoder convolutional neural network (RED-CNN). IEEE Trans. Med. Imaging.

[21] DIRECTIVE 97/43/EURATOM DU CONSEIL du 30 juin 1997 relative à la protection sanitaire des personnes lors d'expositions aux rayonnements ionisants à des fins médicales (1997).

[22] Protection et Sûreté Radiologiques (1996). Publication 73 de la Commission Internationale de Protection Radiologique (CIPR).

[23] McNitt-Gray, M F. “AAPM/RSNA Physics Tutorial for Residents: Topics in CT.” Radiographics 22, no. 6 (January 1, 2002): 1541. 10.1148/rg.226025128 12432127

[24] Claudia Z (2013). Pediatric CT: strategies to lower radiation dose. AJR Am. J. Roentgenol..

[25] Arrêté du 23 mai 2019 portant homologation de la décision n° 2019-DC-0667 de l'Autorité de sûreté nucléaire du 18 avril 2019 relative aux modalités d'évaluation des doses de rayonnements ionisants délivrées aux patients lors d'un acte de radiologie, de pratiques interventionnelles radioguidées ou de médecine nucléaire et à la mise à jour des niveaux de référence diagnostiques associés. NOR: SSAP1915191A (2019).

[26] Hollingsworth C, Frush DP, Cross M, Lucaya J (2003). Helical CT of the body: a survey of techniques used for pediatric patients. Am. J. Roentgenol..

[27] Singh S (2009). Dose reduction and compliance with pediatric CT protocols adapted to patient size, clinical indication, and number of prior studies. Radiology.

[28] Strauss KJ (2010). Image gently: ten steps you can take to optimize image quality and lower CT dose for pediatric patients. Am. J. Roentgenol..

[29] Moifo B (2017). Diagnostic reference levels of adults ct-scan imaging in cameroon: a pilot study of four commonest ct-protocols in five radiology departments. Open J. Med. Imaging.

[30] IRSN. *Doses délivrées aux patients en scanographie et en radiologie conventionnelle*. Résultats d’une enquête multicentrique en secteur public. Rapport DRPH/SER N°2010–12. Direction de la radioprotection de l’homme. Service d’Etudes et d’Expertises en Radioprotection. 13–26 (2010).

[31] Deschênes, S. *Effets de la radiation en imagerie diagnostique.* Reseau mère-enfant Ph. D. Physicien médical, CHU Ste-Justine. sylvain.deschenes@recherchesylvain.recherche-ste-Justine.qc.ca. pp 23–33 (2015).

[32] Donnelly LF (2005). Reducing radiation dose associated with pediatric ct by decreasing unnecessary examinations. Am. J. Roentgenol..

